# Primary care in the Asia-Pacific region: challenges and solutions

**DOI:** 10.1186/1447-056X-11-8

**Published:** 2012-10-05

**Authors:** Richard Hays, Lam Tai Pong, Zorayda Leopando

**Affiliations:** 1 Bond University, Gold Coast, Queensland, Australia; 2 Department of Family Medicine and Primary Care, The University of Hong Kong, Hongkong, Hong Kong; 3 Department of Family and Community Medicine, University of the Philippines Manila, Manila, Philippines

## 

*Asia Pacific Family Medicine* aims to provide a forum for the dissemination of high quality regional research and to enhance the standards of family medicine by focusing on best practice. The ultimate goal is to enhance the provision of primary care to our patients.

## Regional diversity

Geographically large and home to a substantial proportion of the world’s population, the region includes substantial diversity in culture, language, economic maturation and demography. Most nations share problems such as ageing populations, increasing complexity of disease, workforce shortages, a need for increasing technology and accelerating costs. Some project substantial growth in middle class affluence and aspiration, which are major drivers of demand for improved quality. Some still struggle with high child mortality and infectious disease prevalence, particularly with Malaria and Dengue fever. While some show potential to be the economic powerhouses of the near future, a few have substantial poverty, sometimes related to civil warfare and terrorism. Yet these health issues are managed by diverse health care systems, with different proportions of primary, secondary and tertiary care, access to health care and funding models. The Asia-Pacific region shares with the rest of the world the challenges of allocating health care resources to achieve efficiently an increase in the quality of health care. In a sense the region has the potential to be a fascinating laboratory for comparing just how the shared challenges can be managed.

## Healthcare system diversity

There are at least three kinds of health care system within the region: the British legacy of strong, first contact, medical primary care; US-style ‘competition’ between primary and secondary care; and non-medical primary health care, all either with or without universal access. Family Medicine is not always recognized as a defined specialty and, where present, requires variable levels of training and recognition. Health care is often delivered by innovative categories of less trained and less expensive workers. Primary medical care is believed to be associated with more equitable and more cost-efficient health care, and private primary care has been shown to be ‘better’ in Hong Kong when delivered through private rather than public practices [1]. On the other hand, non-medical primary care works well in less developed nations, achieving high vaccination rates and success with other prevention measures. China has a mixed system, but has some of the best health statistics in the world as well as now challenging the USA, UK and Europe for leadership in the advance of medical science, begging the question: how?

## Healthcare system reforms

Major reforms to health systems are under consideration almost everywhere, as costs escalate much faster than growth in national wealth. For example, the USA, home to a bimodal system of both excellent (technical quality of) and poor (access to) health care, wants to extend access. The UK has a universal access system that is struggling to cope due to rapid cost increases. Within the region, Singapore plans to increase access and, in parallel with Malaysia, is seeking to become a health hub for the rest of Asia, perhaps benefiting from medical tourism. Hong Kong is considering increasing the size of the private health system [2]. Australia and New Zealand have universal access systems, but access often requires some co-payment through a form of government-supported public health system.

## Family medicine training

Many countries in the region have formal postgraduate training in Family Medicine but their duration, content and pathways vary [3]. It is not a question of one better than the others. They are established with specific regulations and specifications in mind [4-7]. This also leads to varying levels of recognition by other medical specialties, with specialist status in some countries [8-10]. Furthermore, mandatory referrals from primary care practitioners are not required in some systems within the region. How do these variations affect the interface between primary care and secondary care?

Common mental health disorders e.g. anxiety and depression offers a good example to examine the roles that primary care can play in the region. These high prevalent disorders cannot be dealt with by specialist psychiatrists alone. However, are most primary care professionals competent in dealing with them? What kind of training would help to enhance their skills to a level sufficient for optimal care? Other high prevalent disorders e.g. hypertension and diabetes mellitus offer similar opportunities to examine the role of primary care in different localities.

## Opportunities

The natural experiment we are observing in the Asia-Pacific region suggest several research questions. Which of the three health system models is appropriate for which conditions? Is there a relationship between presence of a recognized Family Medicine specialty and health care quality?

As primary care gets better developed in the region, we must also recognize the different needs of different populations. In countries with established primary care, most of the general population recognize their general practitioners/family physicians as their first contact point with the healthcare system. However, such is not the situation in countries where primary care is relatively unestablished but is being promoted [11-15]. It would also be highly relevant to examine how acceptable Family Medicine is in being the first contact care for the general population: is this about improving access or ‘gate-keeping’?

There is never going to be one model fits all. However, there are certainly lessons and experiences to be shared among the different systems. *Asia Pacific Family Medicine* welcomes submissions from different systems so that advances are made to enhance our service to our patients.

## Competing interests

The authors declare that they have no competing interests.

## Authors’ contributions

The first draft was written by RH. LTP and ZEL reviewed, comments and added a few statements. All authors read and approved the final manuscripts.

## Editors’ note

This is the “Valedictory Remark” by Professor Richard Hays as the outgoing Co Editor-in-Chief of *Asia Pacific Family Medicine.* The Co Editors-in-Chief and the Editorial Board would like to thank Professor Hays for his contributions to the journal since its inception. We would also like to welcome our new Co Editor-in-Chief, Professor Kelsey Hegarty.
